# Effect of preoperative dynamic cervical sagittal alignment on the loss of cervical lordosis after laminoplasty

**DOI:** 10.1186/s12891-023-06335-8

**Published:** 2023-03-28

**Authors:** Chengxin Liu, Bin Shi, Wei Wang, Xiangyu Li, Shibao Lu

**Affiliations:** 1 Department of Orthopedics, Xuanwu Hospital, Capital Medical University, Beijing, China; 2 National Clinical Research Center for Geriatric Diseases, Beijing, China

**Keywords:** Cervical sagittal alignment, Laminoplasty, Loss of cervical lordosis, Flexion and extension function

## Abstract

**Purpose:**

Cervical laminoplasty (CLP) is a developed surgical procedure for the treatment of cervical spondylotic myelopathy (CSM), but only a few of those studies focus on preoperative dynamic cervical sagittal alignment and the study of different degrees of loss of cervical lordosis (LCL) is lacking. This study aimed to analyze patients who underwent CLP to investigate the effect of cervical extension and flexion function on different degrees of LCL.

**Methods:**

In this retrospective case–control study, we analyzed 79 patients who underwent CLP for CSM between January 2019 and December 2020. We measured the cervical sagittal alignment parameters on lateral radiographs (neutral, flexion, and extension positions) and used Japanese Orthopedic Association (JOA) score to assess clinical outcomes. We defined the extension ratio (EXR) as 100 × Ext ROM (cervical range of extension)/ROM (cervical range of motion). We observed the relationships between collected variables (demographic and radiological variables) and LCL. Patients were classified into the following three groups according to the LCL: stability group: (LCL ≤ 5°); mild loss group (5° < LCL ≤ 10°); and severe loss group (LCL > 10°). We compared the differences of collected variables (demographic, surgical and radiological variables) among the three groups.

**Results:**

Seventy-nine patients were enrolled (mean age 62.92 years; 51 men, 28 women) in the study. Among the three groups, cervical Ext ROM was the best in the stability group (*p* < 0.01). Compared with the stability group, range of flexion (Flex ROM) was significantly higher (*p* < 0.05) and EXR was significantly lower (*p* < 0.01) in the severe loss group. Compared with the severe loss group, JOA recovery rates were better (*p* < 0.01) in the stability group. Receiver-operating characteristic curve (ROC) analysis to predict LCL > 10° (area under the curve = 0.808, *p* < 0.001). The cutoff value for EXR was 16.80%, with sensitivity and specificity of 72.5% and 82.4%, respectively.

**Conclusion:**

CLP should be carefully considered for patients with a preoperative low Ext ROM and high Flex ROM, as a significant kyphotic change is likely to develop after surgery. EXR is a useful and simple index to predict significant kyphotic changes.

## Introduction

Cervical spondylotic myelopathy (CSM) is one of the most prevalent neurological disorders in older population which is characterized by neck pain, and sensory, motor, and/or reflex deficits [1]. Cervical laminoplasty (CLP) is a developed surgical procedure for the treatment of CSM [2–5]. CLP can preserve the motion of the operated levels and is more suitable for patients undergoing a multi-segmental cervical spinal cord surgery. However, cervical laminoplasty, as posterior non-fusion decompression surgery, can lead to some potential complications, such as loss of cervical lordosis (LCL), decreased neck motion range, axial neck pain, C5 nerve root palsy, and lamina closure. Among them, LCL is a significant issue.

The alignment of the cervical spine needs to maintain adequate lordosis to provide enough space for the shifting of the spinal cord. Therefore, prevention of LCL and investigation of its risk factors after CLP are necessary. Several studies have reported many preoperative predictors of LCL after laminoplasty [6–21]. and the relationship between static cervical sagittal alignment and kyphotic changes has been well described [6–9, 13, 15, 17, 18, 20–22]. However, only a few of those studies focus on preoperative dynamic cervical sagittal alignment [11, 14, 16] and the study of different degrees of LCL is lacking.

We aimed to retrospectively analyze patients who underwent laminoplasty to investigate the effect of preoperative static and dynamic cervical sagittal alignment on LCL after laminoplasty. We hypothesized preoperative cervical extension function and flexion function were associated with different degrees of LCL and neurological recovery and found out potential risk factors for serious post-laminoplasty LCL.

## Materials and methods

### Patient enrollment

This study was approved by the IRB of our affiliated institution (IRB number: 2018-086). This was a retrospective case–control study of the patients who underwent cervical laminoplasty at our institution between January 2019 and December 2020. Patients were eligible for our study if they met the following inclusion criteria: 1) aged 18 years or older without previous cervical surgery; 2) a lesion involving more than three levels of CSM 3) clinical signs of myelopathy (difficulty with manual dexterity, upper extremity numbness, gait disturbance); and 4) at least twelve months of follow-up. Patients with fractures, infections, tumors, combinations with fusion surgery, decompression levels including C2 or thoracic spine levels, and invisible T1 upper endplates were excluded. Finally, seventy-nine patients were enrolled.

### Surgical procedures

After the induction of general endotracheal anesthesia, patients were positioned prone on the operating table. The surgeons performed an incision in the back of the neck, and detached the paravertebral muscle from the spinous process and lamina, preserving the facet capsule. All patients underwent open-door laminoplasty with a mini titanium plate system for decompression. One side of the lamina was opened, and the other side served as the hinge.

### Radiological parameters

**Fig. 1:**
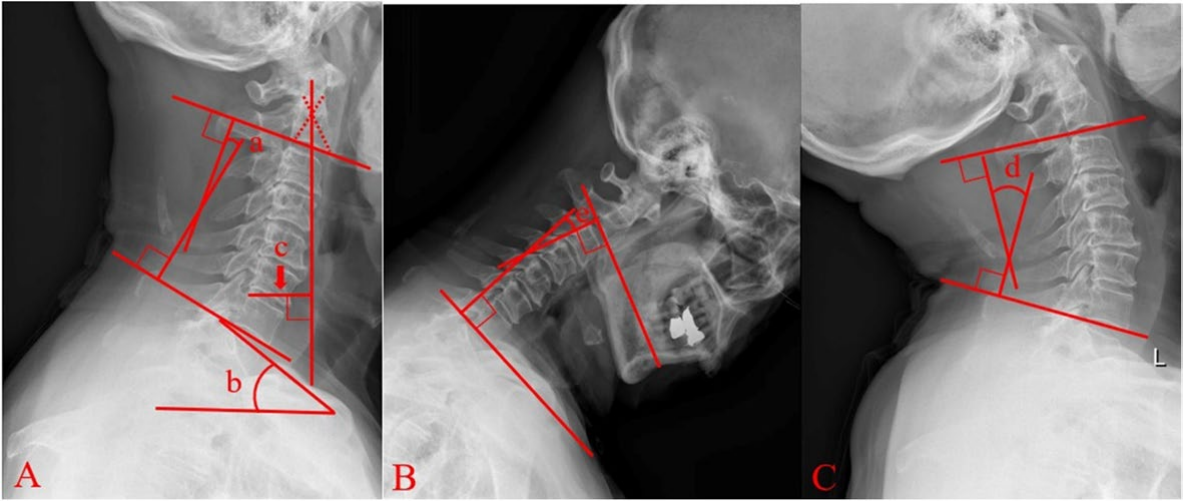
CL (a), T1S (b), and cSVA (c) measured in the neutral position. Flex CL (d) and Ext CL (e) measured with the patient in maximal flexion and extension, respectively. CL, cervical lordosis; T1S, T1 slope; cSVA, cervical sagittal vertical axis; Flex CL, CL in flexion; Ext CL, CL in extension

The cervical sagittal alignment parameters were measured on lateral radiographs (Fig. 1): cervical lordosis (CL) was the angle between the C2 lower endplate and the C7 lower endplate; T1 slope (T1S) was the angle between a horizontal plane and a line parallel to the superior T1 endplate; cervical sagittal vertical axis (cSVA) was defined as the horizontal offset from a plumbline dropped from the C2 vertebral body to the posterosuperior corner of the C7 vertebra; CL in flexion (Flex CL) and extension (Ext CL) were measured on radiographs in the flexion and extension positions.

**Fig. 2:**
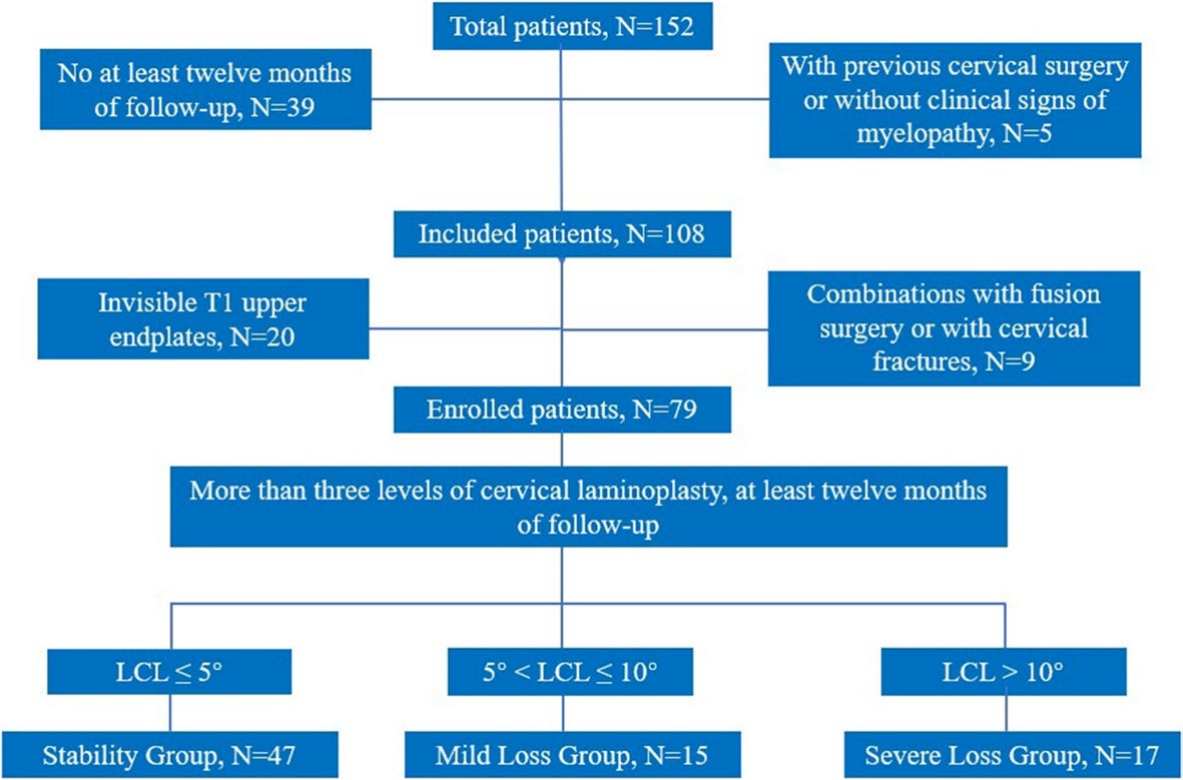
The flowchart of study. LCL: loss of cervical lordosis

The cervical spine range of motion (ROM) was calculated as Ext CL—Flex CL. Cervical spine range of flexion (Flex ROM) was calculated as CL—Flex CL and cervical spine range of extension (Ext ROM) was calculated as Ext CL—CL. The extension ratio (EXR) was defined as 100 × Ext ROM/ROM. LCL was defined as preoperative CL—postoperative CL. Patients were classified into the following three groups according to the LCL [11, 21]: stability group: (LCL ≤ 5°); mild loss group (5° < LCL ≤ 10°); and severe loss group (LCL > 10°). The flowchart of study is displayed in Fig. 2.

### Clinical parameters

The Japanese Orthopedic Association (JOA) score [23], before surgery and at the 1-year follow-up visit, was used to evaluate clinical outcomes. The recovery rate was calculated as follows: JOA recovery rate = 100 × (postoperative JOA—preoperative JOA) / (17—preoperative JOA).

### Statistical analysis

All the data were analyzed using SPSS version 22.0 software (SPSS, Inc, Chicago, IL, USA). Variables were described as mean ± standard deviation and interclass correlation coefficient was used to indicate the measurement consistency between two observers. Pearson correlation analysis was used to analyze the correlation; multiple linear regression model was used to explore the risk factors for LCL. Chi-square test was used to compare categorical data among the groups; T-tests, ANOVA, and Kruskal–Wallis tests were used to assess the differences of radiographic parameters among the groups. A receiver-operating characteristic curve (ROC) analysis was used to determine the optimal cutoff value. *P* value < 0.05 was considered as evidence of statistical significance.

## Results

Seventy-nine patients were enrolled (mean age 62.92 years; 51 men, 28 women) in the study. CL decreased significantly after CLP (pre-, 17.34 ± 10.44 vs. post 12.37 ± 11.42, *p* < 0.01). The overall demographic, surgery segments, and proximal level are summarized in Table 1.

**Table 1:** Summary of patient population and LCL correlations (*N* = 79)

**Demographic**	
**Sex**	
Male	51 (64.57%)
Female	28 (35.43%)
**BMI **(**kg/m**^**2** ^**)**	
	25.23 ± 3.87
**CL (°)**	
Pre	17.34 ± 10.44
Post	12.37 ± 11.42
*P* value	< 0.01
**JOA**	
Pre	12.66 ± 2.26
Post	15.20 ± 1.11
Recovery rate (%)	57.95 ± 23.28
**Surgery segment (n)**	
3	29
4	36
5	14
**Proximal level**	
C3	49
C4	30

### Correlations between LCL and preoperative parameters

In the correlation analysis, LCL was positively correlated with cervical flexion capacity (*r* = 0.278, *p* < 0.05) and negatively correlated with cervical extension capacity (*r* = -0.456, *p* < 0.01). No significant correlations were observed between the other evaluated parameters (Table 2).

**Table 2:** LCL correlations (*N* = 79)

**Parameter**	**Mean ± SD**	**Pearson**
**Age (y)**	62.92 ± 9.97	0.081
**Follow-up period (months)**	19.69 ± 8.72	0.119
**Pre CL (°)**	17.34 ± 10.44	0.212
**Pre T1S (°)**	29.35 ± 7.43	0.205
**Pre cSVA (mm)**	22.21 ± 12.65	-0.175
**T1S-CL (°)**	12.01 ± 8.41	-0.082
**Flex CL (°)**	-13.33 ± 8.81	-0.128
**Ext CL (°)**	26.78 ± 11.65	-0.053
**Total ROM (°)**	42.09 ± 12.58	0.041
**Flex ROM (°)**	32.66 ± 12.02	0.278*
**Ext ROM (°)**	9.43 ± 6.21	-0.456**

^*^p < 0.05, **p < 0.01statistically significant difference

### Risk factors for LCL

Multiple linear regression analysis was conducted by using variables that were found to be significantly correlated with the LCL. The results suggested that LCL decreased by 0.421° (*p* < 0.001) for each extension CL, and increased by 0.208° (*p* = 0.042) for flexion CL. LCL could be predicted by using the following regression equation: LCL = 5.507—0.421 * Ext ROM + 0.208 * Flex ROM (Table 3).

**Table 3:** Multiple linear regression model shows correlations between the loss of cervical lordosis and preoperative parameters (*N* = 79)

**Model**	**Unstandardized coefficients**		**Standardized coefficient**	**T**	**Sig**
	**B**	**SE**	**β**		
(Constant)	5.507	2.562		2.105	0.035
Ext ROM (°)	-0.498	0.119	-0.421	-4.184	0.000
Flex ROM (°)	0.127	0.061	0.208	2.069	0.042

### Comparison of evaluated parameter variables according to postoperative LCL

Compared with the stability group (LCL ≤ 5°), the preoperative CL was significantly higher while postoperative CL was significantly lower in the severe loss group (LCL > 10°). Among the three groups, cervical extension Ext ROM was the best in the stability group. Compared with the stability group, Flex ROM was significantly higher and the extension ratio (EXR) was significantly lower in the severe loss group. As for clinical symptoms, pre- and postoperative JOA did not significantly differ among the three groups. Compared with the severe loss group, JOA recovery rates were better in the stability group (Table 4).

**Table 4:** Comparison of evaluated parameters variable according to the postoperative loss of CL

**Parameter**	**Stability group** **LCL ≤ 5°** **(** ***n*** ** = 47)**	**Mild loss group** **5° < LCL ≤ 10°** **(** ***n*** ** = 15)**	**Severe loss group** **LCL > 10°** **(** ***n*** ** = 17)**	***P***
**Age (y)**	62.34 ± 10.49	66.47 ± 7.32	61.41 ± 10.29	*p* > 0.05
**Follow-up period (months)**	18.77 ± 7.51	20.88 ± 9.54	20.71 ± 11.03	*p* > 0.05
**Surgery segment**	3.89 ± 0.72	3.53 ± 0.64	3.82 ± 0.73	*p* > 0.05
**Proximal level (C3)**	28/47	9/15	12/17	*p* > 0.05
**Pre CL (°)**	15.92 ± 9.63^*^	16.93 ± 10.49	23.31 ± 11.42^*^	***p*** ** < 0.05**
**Pre T1S (°)**	28.59 ± 6.65	28.79 ± 8.58	31.95 ± 8.28	*p* > 0.05
**Pre cSVA (mm)**	24.62 ± 13.33	20.44 ± 9.57	17.11 ± 11.86	*p* > 0.05
**T1S-CL (°)**	12.92 ± 8.01	11.85 ± 7.58	9.65 ± 10.09	*p* > 0.05
**Flex CL (°)**	-14.95 ± 8.32	-16.76 ± 8.19	-15.08 ± 10.87	*p* > 0.05
**Ext CL (°)**	27.37 ± 11.73	24.45 ± 11.32	27.19 ± 12.14	*p* > 0.05
**Post CL (°)**	15.29 ± 9.69^@^	9.40 ± 11.49	6.48 ± 13.52^@^	***p*** ** < 0.01**
**Total ROM (°)**	42.32 ± 12.98	41.20 ± 8.64	42.27 ± 14.86	*p* > 0.05
**Flex ROM (°)**	30.63 ± 11.43^*^	33.69 ± 9.80	37.39 ± 14.40^*^	***p*** ** < 0.05**
**Ext ROM (°)**	11.69 ± 5.99^*@^	7.51 ± 5.66^*^	4.88 ± 4.00^@^	***p*** ** < 0.05**
**EXR (%)**	28.25 ± 14.58^@^	20.65 ± 13.98	11.79 ± 9.22^@^	***p*** ** < 0.01**
**Pre JOA**	12.43 ± 2.88	12.87 ± 1.73	13.13 ± 1.00	*p* > 0.05
**Post JOA**	15.57 ± 1.13	14.86 ± 1.19	14.50 ± 1.01	*p* > 0.05
**JOA recovery rate (%)**	69.23 ± 20.34^@^	48.18. ± 30.54	35.40 ± 25.01^@^	***p*** ** < 0.01**

^*^Indicated *p* value ≤ 0.05 and @ indicated *p* value ≤ 0.01

### Effectiveness of EXR to predict severe lordosis loss

**Fig. 3:**
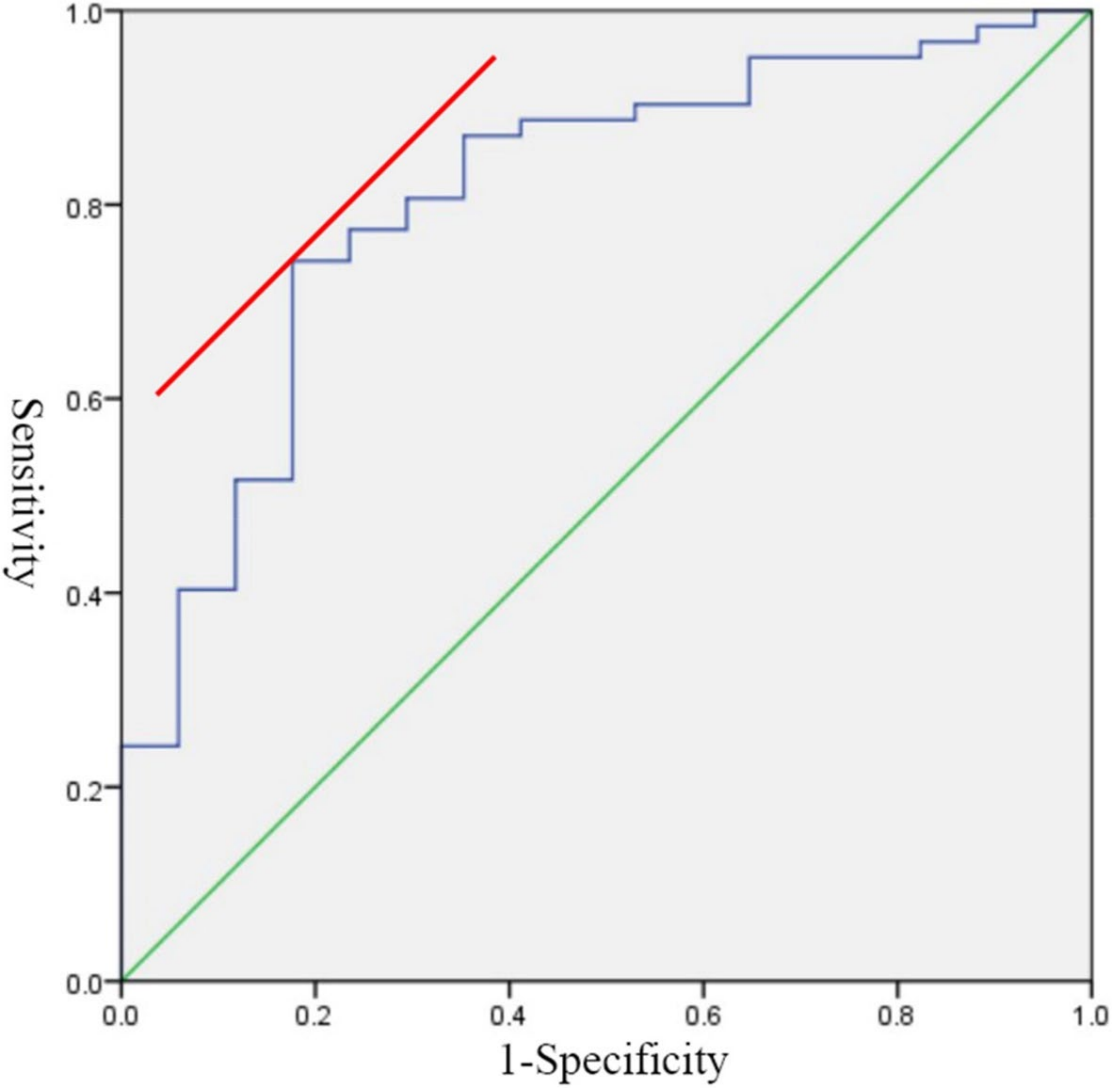
ROC curve analysis to predict LCL > 10° (area under the curve = 0.808, p < 0.001). The cutoff value for EXR was 16.80%, with a sensitivity of 72.5% and a specificity of 82.4%. CL, cervical lordosis; LCL, preoperative CL—postoperative CL; ROC, receiver-operating characteristic

The ROC curve in Fig. 3 shows good discriminative power of EXR to predict severe lordosis loss after CLP (area under the curve = 0.808, *p* < 0.001; cutoff value, 16.80%; sensitivity: 72.5%; specificity: 82.4%; positive predictive value: 60.0%; negative predictive value: 96.3%). In the severe loss group, 15/17 patients were subclassified as low EXR (EXR ≤ 16.80%); in the mild loss group, 6/15 patients were subclassified as the low EXR group. In the stability group, only 4/47 patients were subclassified into the low EXR group.

## Discussion

Having sufficient postoperative cervical lordosis is a prerequisite for CLP to obtain the indirect anterior decompression effect. LCL has been reported to be associated with poor outcomes after laminoplasty in many studies [7–9, 17, 24, 25]. Kim et al. [17] and Miyazaki et al. [19] reported that preoperative higher T1S was a risk factor for LCL. T1S-CL [8, 17, 22] and CL/T1S [26] have also been considered as predictors for LCL after CLP. However, there are some studies showing different results regarding the correlation between T1S and LCL [16, 27–29]. Michael et al. [12] and Seo et al. [8] emphasize the importance of cephalad vertebral level and cervical foraminal stenosis in LCL after laminoplasty. Some studies also report other factors for LCL, such as cSVA [15, 17, 22], C7-SVA [20, 21], CGH-C7 SVA [7, 9] and age [6]. In the present study, we evaluated regional static parameters to identify possible risk factors for postoperative kyphotic alignment change. However, no significant correlations were observed between the static parameters and LCL in our study. These static parameters have their own limitations in accounting for LCL after surgery. According to our knowledge, there are no theories with consensus that explain why these static parameters can affect LCL after surgery. Many researchers think that the posterior neck muscular-ligament complex may play an important role in these processes [6, 12, 16–18, 22, 30, 31].

Recently, some studies have shown the relationship between preoperative dynamic cervical sagittal alignment and LCL after CLP. Lee et al. [16] reported the extension function of the cervical spine as an indicator to predict kyphotic change after CLP, and showed that significant kyphotic change occurred in patients whose Ext ROM was < 14°. Moreover, some studies have shown that higher Flex ROM results in greater LCL after CLP [11, 18, 30–32]. The present study showed similar results to those studies: preoperative Ext ROM (β = -0.421) and Flex ROM (β = 0.208) were predictors for postoperative LCL. Our study reported a high negative correlation between Ext ROM and LCL, which implies that enough Ext ROM is a highly reliable factor in preventing LCL after CLP. Similar to the results of previous studies [11, 18, 30–32], our study shows the positive correlation between Flex ROM and LCL. Cervical flexion mobility is blocked by degenerative structures, such as bone, ligaments, or muscles. Fujishiro et al. [30] speculated that increased motion in the flexional direction indicates that such structural forces restricting motion toward the kyphotic position are weak. Because of the surgical injury, the equilibrium necessary to maintain cervical sagittal alignment is disrupted and results in a higher prevalence of LCL.

**Fig. 4:**
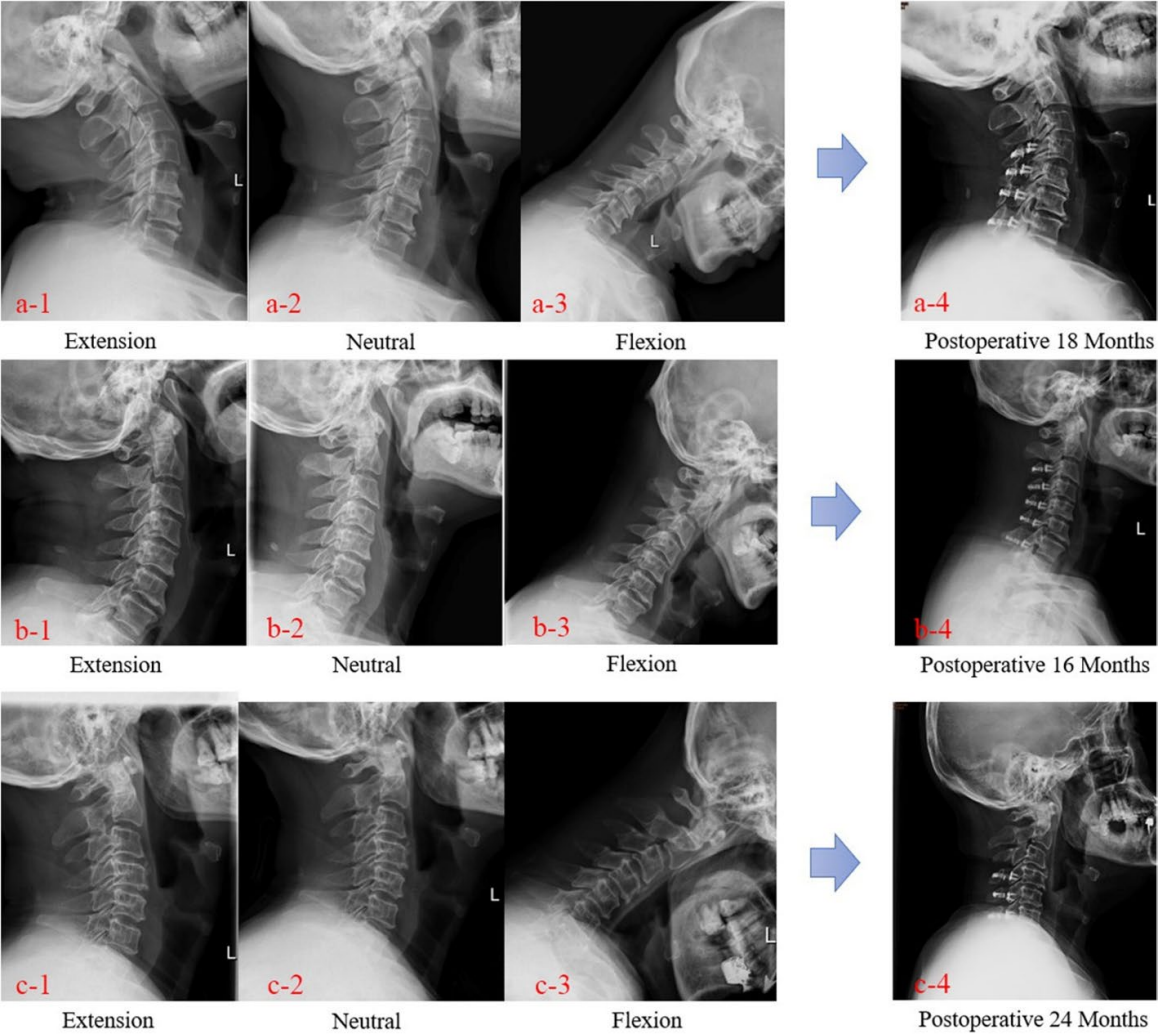
Representative cases. Sagittal preoperative radiographs (a-1, 2, 3) showing increased Ext ROM (20.3°) in a 65-year-old man who underwent laminoplasty of C4–7; no significant LCL was observed 18 months after surgery (a-2, a-4: pre CL 20.1° vs. post CL 24.9°). Sagittal preoperative radiographs (b-1, 2, 3) showing low Ext ROM (8.2°) and Flex ROM (29°) in a 48-year-old man who underwent laminoplasty of C3–7; mild LCL was observed 16 months after surgery (b-2, b-4: pre CL 16.2° vs. post CL 8.6°). Sagittal preoperative radiographs (c-1, 2, 3) showing low Ext ROM (3.1°) and increased Flex ROM (38.3°) in a 55-year-old man who underwent laminoplasty of C4–6; severe LCL was observed 24 months after surgery (c-2, c-4: pre CL 15.4° vs. post CL -7.6°). CL, cervical lordosis; LCL, loss of cervical lordosis; Flex ROM, cervical spine range of flexion; Ext ROM, cervical spine range of extension

**Fig. 5:**
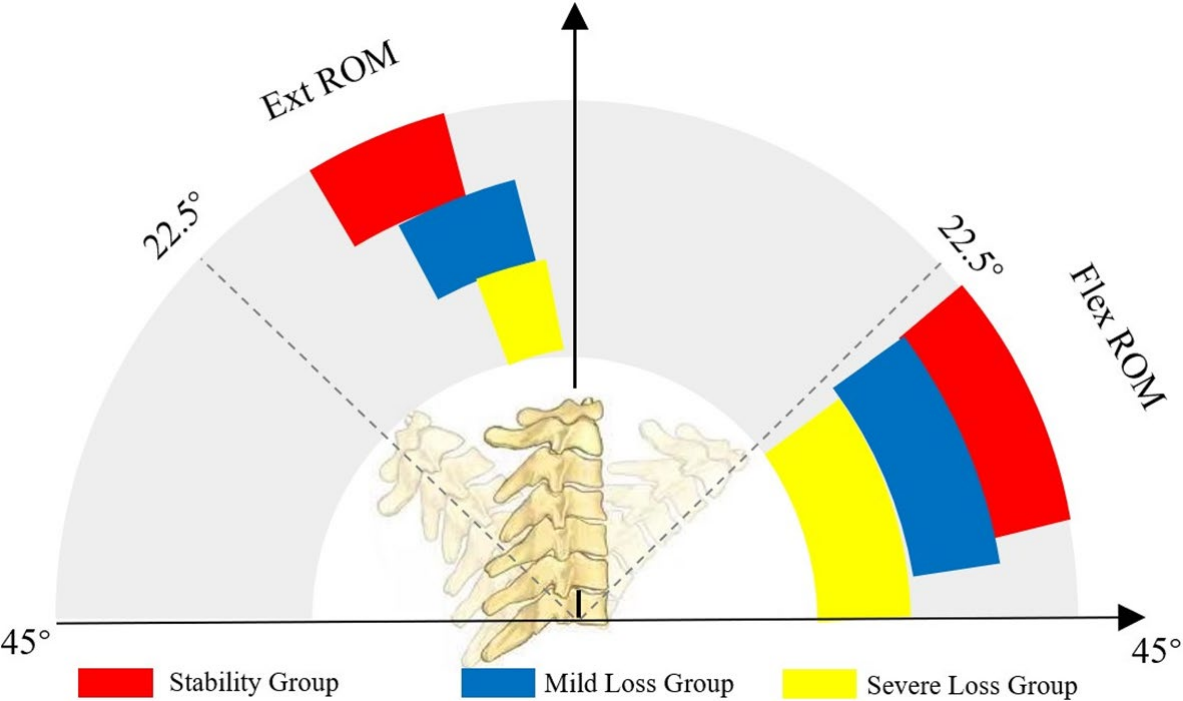
Ranges of Ext ROM and Flex ROM (25% quartile-75% quartile). The ranges of Ext ROM in the three groups were 7–15°; 4–11.5°; and 2–8°, respectively. The ranges of Flex ROM in three groups were 23–37°; 23–41°; and 27–46°, respectively. Flex ROM, cervical spine range of flexion; Ext ROM, cervical spine range of extension

In our study, we discovered that different degrees of postoperative LCL implied different degrees of neurological recovery. Worse JOA recovery rate was reported in patients in the severe loss group compared with the stability group. Similar tendencies were also shown between the stability group and the mild loss group; however, there was no evidence of statistical significance (*p* > 0.05). Postoperative mild LCL occurred in patients with a low level of Ext ROM and the influences of LCL on postoperative neurological recovery were limited. Preoperative high levels of Flex ROM aggravate postoperative LCL for patients with low Ext ROM, and severe LCL implies poor clinical outcomes (Table 4, Figs. 4 and 5).

Some researchers have speculated that the degree of cervical extension mobility indicates the cervical constriction reservoir [16] and cervical flexion mobility indicates the forces inhibiting cervical kyphosis [30]. Both Flex ROM and Ext ROM were important factors for LCL after CLP. Ono et al. [11] proposed CL ratio (100 × Flex ROM / total ROM) as a novel predictor for the loss of cervical lordosis after laminoplasty and reported the cut off value of CL ratio for predicting postoperative LCL. Compared with Flex ROM, Ext ROM had a greater influence on postoperative LCL in our study. Therefore, we reported EXR (100 × Ext ROM / total ROM) as a predictor, and EXR showed better prediction in severe lordosis loss than Ext ROM or Flex ROM alone. The optimal cutoff value of EXR to discriminate between severe LCL and not severe LCL was 16.8% (Fig. 3). For patients with a preoperative EXR less than 16.8%, more cervical exercises should be encouraged after surgery due to the high prevalence of severe postoperative LCL. Multilevel posterior cervical fusion or anterior cervical fusion surgery can also be considered, if necessary.

This study has several limitations. First, because our study was retrospective, a selection bias may exist. Second, the number of patients was low. Only 17 cases were assigned to the severe loss group. Third, the follow-up period was 1 year. Choi et al. [33] reported that changes in cervical sagittal alignment generally reach a plateau at 6 months after CLP. Thus, the follow-up period was enough to investigate the risks for LCL after CLP. Finally, only the JOA score was used to evaluate clinical outcomes in the present study.

## Conclusion

Preoperative dynamic cervical sagittal alignment is a highly useful indicator to predict the LCL after CLP. CLP should be carefully considered for patients with a preoperative low Ext ROM and high Flex ROM, as a significant kyphotic change is likely to develop after surgery. EXR is a useful and simple index to predict significant kyphotic changes.

## Data Availability

The datasets generated and/or analysed during the current study are not publicly available due [This study is part of a series of studies that have not been completely completed] but are available from the corresponding author on reasonable request.

## Abbreviations

CLP: Cervical laminoplasty
CSM: Cervical spondylotic myelopathy
LCL: Loss of cervical lordosis
CL: Cervical lordosis
T1S: T1 slope
cSVA: Cervical sagittal vertical axis
Flex CL: Cervical lordosis in flexion
Ext CL: Cervical lordosis in extension
ROM: Cervical spine range of motion
Flex ROM: Cervical spine range of flexion
Ext ROM: Cervical spine range of extension
EXR: Extension ratio
JOA: Japanese Orthopedic Association
ROC: Receiver-operating characteristic curve
